# Development and validation of cuproptosis-related gene signature in the prognostic prediction of liver cancer

**DOI:** 10.3389/fonc.2022.985484

**Published:** 2022-08-12

**Authors:** Yanqing Liu, Yang Liu, Shujun Ye, Huijin Feng, Lianjun Ma

**Affiliations:** ^1^ Endoscopy Center, China-Japan Union Hospital of Jilin University, Changchun, China; ^2^ Herbert Irving Comprehensive Cancer Center, Columbia University, New York, NY, United States

**Keywords:** liver cancer, cuproptosis, lncRNA, mRNA, prognostic model

## Abstract

Liver cancer is a generic term referring to several cancer types arising from the liver. Every year, liver cancer causes lots of deaths and other burdens to the people all over the world. Though the techniques in the diagnosis and therapy of liver cancer have undergone significant advances, the current status of treating liver cancer is not satisfactory enough. The improvement of techniques for the prognosis of liver cancer patients will be a great supplement for the treatment of liver cancer. Cuproptosis is a newly identified regulatory cell death type, which may have a close connection to liver cancer pathology. Here, we developed a prognostic model for liver cancer based on the cuproptosis-related mRNAs and lncRNAs. This model can not only effectively predict the potential survival of liver cancer patients, but also be applied to evaluate the infiltration of immune cell, tumor mutation burden, and sensitivity to anti-tumor drugs in liver cancer. In addition, this model has been successfully validated in lots of liver cancer patients’ data. In summary, we wish this model can become a helpful tool for clinical use in the therapy of liver cancer.

## Introduction

Liver cancer is one of the major cancer types and among the most malignant liver diseases around the world (1–5). Liver cancer comprises several sub-types, including hepatocellular carcinoma (the most common type of liver cancer), cholangiocarcinoma, hepatoblastoma, and angiosarcoma (1). Liver cancer causes great healthy and economic burdens to the people and the society of both developed and underdeveloped area. According to the estimation of epidemiologists, there were about 905,677 new cases of liver cancer and 830,180 new deaths caused by it (2). In the US, although liver cancer is not among the top 10 cancer types regarding the estimated new cases in 2022, it may cause more than 30,000 deaths in the same year, which ranks 5^th^ in all the cancer types (6). Albeit the dramatic development in the diagnosis and treatment of liver cancer in the past decades, its mortality increases rapidly, partially due to the change of environment, life style, and dietary habit (1, 7, 8). For scientific and clinical researchers, elucidating the underlying mechanism for the initiation and development of liver cancer is a crucial and long-term task. In the meantime, development of novel prognostic biomarker for liver cancer patients will greatly benefit their treatments (1, 9).

For the somatic cells in mammalian life, the equilibrium between cell death and proliferation is of vital importance. To achieve a concerted life circle, human cells “master” multiple regulated cell death (RCD) modalities (including apoptosis, ferroptosis, necroptosis, and pyroptosis) evolutionally (10). During normal development, these RCDs cooperate with each other to orchestrate a suitable rate of cell turnover in different organs. In addition, these RCDs also function in dealing with various inner or environmental stresses. From a clinical perspective, these RCDs have been demonstrated to be involved with a wide range of disease processes, including organ injury, immune system dysfunction, neurodegenerative disorder, and particularly tumor (10). Identifying novel RCD type will not only deepen our understanding of the human cell, but also create new therapeutic opportunity for lots of diseases, including liver cancer. Cuproptosis is a quite recently discovered RCD mode, which is characterized by unique features different from other RCDs mentioned above (11). Cuproptosis is caused by copper-induced aggregation of lipoylated proteins in TCA cycle. The data in the seminal paper suggested that cuproptosis may participate in the modulation of various diseases, including cancer. Whether cuproptosis is associated with liver cancer has not been investigated. However, it is of evident significance to explore the potential link between liver cancer and cuproptosis (and essential genes underlying it).

Long non-coding RNA (lncRNA) is one of the research hotspots in these years (12). This is a family of RNAs with diverse lengths, localizations, structures, and functions. The dysregulation of lncRNAs have been demonstrated to be involved in the progression of various tumor types, including liver cancer (13). These lncRNAs can not only be therapeutic targets in liver cancer treatment, but may also constitute unique expression profiles to indicate distinct characteristics of liver cancer, including the malignant stage, the sensitivity to therapeutics, and the prognosis of patients (14). Particularly, differential lncRNA expression profiles can be established based on specific cellular processes. Constructing a solid lncRNA signature in the context of a crucial activity in liver cancer cell will be of great use for predicting the development of liver cancer in patients.

In this study, we leveraged our current knowledge about liver cancer, cuproptosis, and lncRNA to construct a novel prognostic model based on cuproptosis-related lncRNAs and mRNAs in liver cancer. This model fits well with diverse pathologic parameters of liver cancer. Moreover, this model has been successfully validated by using a batch of patients’ data. We believe that, this model can be beneficial to the prognosis of liver cancer patients.

## Materials and methods

### Microarray data

We obtained the gene expression profile, survival information and clinical characteristics of liver cancer patients from TCGA database (https://cancergenome.nih.gov/). A total of 424 samples were used in this study, including 374 liver cancer samples and 50 non-tumor tissues. The mRNAs related to cuproptosis obtained from previous literatures are summarized in **Table S1** (11, 15–19). Pearson correlation analysis was used to identify cuproptosis-related lncRNAs, and the co-expression networks of lncRNAs-mRNAs were established and visualized with R package “ggalluvial”.

### Construction of cuproptosis-related prognostic signature for liver cancer

Univariate Cox regression analysis was used to screen cuproptosis-related mRNAs and lncRNAs that were closely associated with survival. Subsequently, mRNAs and lncRNAs with statistically significant difference (p<0.01) in univariate Cox regression analysis were selected for multivariate Cox regression analysis to determine the potential optimal cuproptosis-related prognostic genes. Based on the prognostic potential and the regression coefficient, the 9-gene signature was finally developed. Next, risk score is calculated according to the formula: Risk score = (exprgene1 × Coefgene1) + (exprgene2 × Coefgene2) + … + (exprgenen × Coefgenen).

### Evaluation of the 9-gene signature including 3 mRNAs and 6 lncRNAs

Median of risk score is used to divide patients into two groups (high and low risk, respectively). Kaplan-Meier survival analysis was performed with “survival” and “survminer” R software packages. ROC curve was performed to calculate the area under the curve (AUC) to evaluate the diagnostic value of the 9-gene signature. Then, C-index curve were used to estimate the model accuracy.

### Construction of nomogram

We used the clinical features (including age, gender, grade and stage) to establish nomograms for predicting survival in patients with liver cancer. In addition, to assess the consistency between predicted and actual survival, calibration curves were drawn.

### Correlation analysis between distinct groups and clinical characteristics

To further explore the correlation between risk scores and clinical characteristics, the distribution of clinicopathological features in differential groups was displayed by R software package “pheatmap”.

### Validation of the model in GEO dataset

1. Spearman correlation analysis was used to screened mRNAs correlated with 9 signature genes (coefficients > 0.40, p < 0.001).
2. Differential expression analysis was performed to classify mRNAs into gene up-regulated cluster A and down-regulated cluster B.
3. The Gene set variation analysis (GSVA) was employed to calculate enrichment score of cluster A and cluster B. Then, we calculated RS score equivalent to subtraction of the enrichment score of cluster B from the enrichment score of cluster A.
4. After calculating the RS scores in each GEO sample, Kaplan-Meier curve was used to evaluate the difference of OS between high RS score group and low RS score group.

### Enrichment functional analysis

We first determined the expression of a set of differentially expressed genes (DEGs) containing mRNAs and lncRNAs between the high-risk group and the low-risk group. The cutoff criteria were FDR < 0.05 and |logFC| > 1. Then go function enrichment analysis and KEGG pathway analysis were performed for DEGs.

### Immune-related functional analysis

To explore the relationship between risk scores and infiltration of immune cell, we quantified the abundance of immune cells in the two risk groups using algorithms such as TIMER, CIBERSORT, and others. In addition, the ssGSEA algorithm was applied to assess the immune-related functions in two risk groups. Besides, referring to existing studies, the expression level of immune checkpoint related genes may be correlated to the clinical efficacy of immune checkpoint inhibitor blockade therapy (20). Therefore, the correlation between risk scores and immune checkpoints was also studied.

### Relationship between hypoxia-related genes and risk score

Hypoxia can regulate TCA cycle and may involve in cuproptosis initiation (21). Therefore, we studied the correlation between expression of hypoxia related genes and risk score.

### Analysis of tumor mutation burden and drug sensitivity

According to the somatic mutation data of each tumor, TMB was calculated as the mutation bases per million bases. The maftools package was used to aggregate and visualize mutation data and evaluate the relationship between risk score and Tumor mutation burden (TMB). Tumor immune dysfunction and Exclusion (TIDE) algorithm was used to predict the immune response. Next, the “pRRophetic” software package of R software was used to evaluate the sensitivity of chemotherapy drugs with the half maximum inhibitory concentration (IC50).

## Results

### Identification of cuproptosis-related LncRNAs and construction of the 9-gene signature

**Figure 1:**
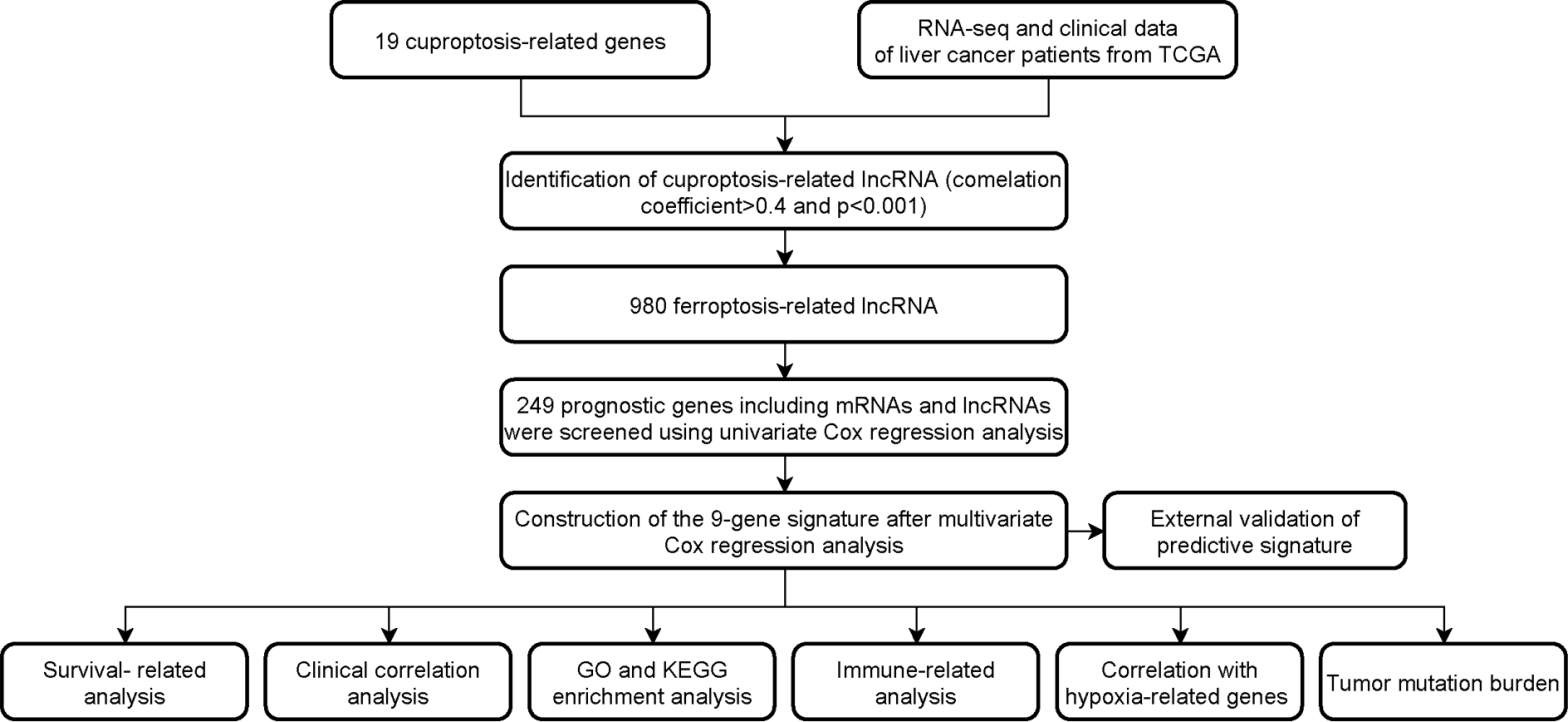
Workflow of the study design.

**Figure 2:**
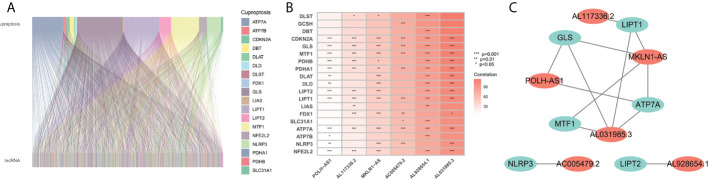
Screening of prognosis-related genes. **(A)** Sankey diagram of the associations between between cuproptosis-related lncRNAs and mRNAs. **(B)** The correlation heatmap of 6 screened lncRNAs and cuproptosis-related genes. **(C)** The network diagram of 6 screened lncRNAs and cuproptosis-related genes.

The flowchart of the research is shown in **Figure 1**. Firstly, 980 cuproptosis-related lncRNAs were identified from 16,773 lncRNAs based on the filtering criteria of a correlation coefficient <0.4 and p < 0.001. The co-expression relationship between cuproptosis-related lncRNAs and cuproptosis-related mRNAs was shown using Sankey diagram (**Figure 2A**). In the TCGA set, univariate Cox regression analysis was used to screen 249 prognostic genes including mRNAs and lncRNAs associated with cuproptosis from the 999 cuproptosis-related genes. Performing Lasso Cox regression analysis and multivariate Cox regression analysis on the TCGA set, we identified robust 9 cuproptosis-related genes containing 3 mRNAs and 6 lncRNAs. The correlation between 6 screened lncRNAs and cuproptosis-related genes was shown in the correlation heatmap and network diagram (**Figures 2B, C**). As a result, the risk score model was constructed as follows: risk score = (-0.1369×ATP7A expression) + (0.0174×DLAT expression) + (-0.0124×GLS expression) + (0.2669×POLH-AS1 expression) + (0.0868×AL117336.2 expression) + (0.3621×MKLN1-AS expression) + (0.2207×AC005479.2 expression) + (0.1527×AL928654.1 expression) + (0.1200×AL031985.3 expression).

### Evaluation of the 9-gene signature

**Figure 3:**
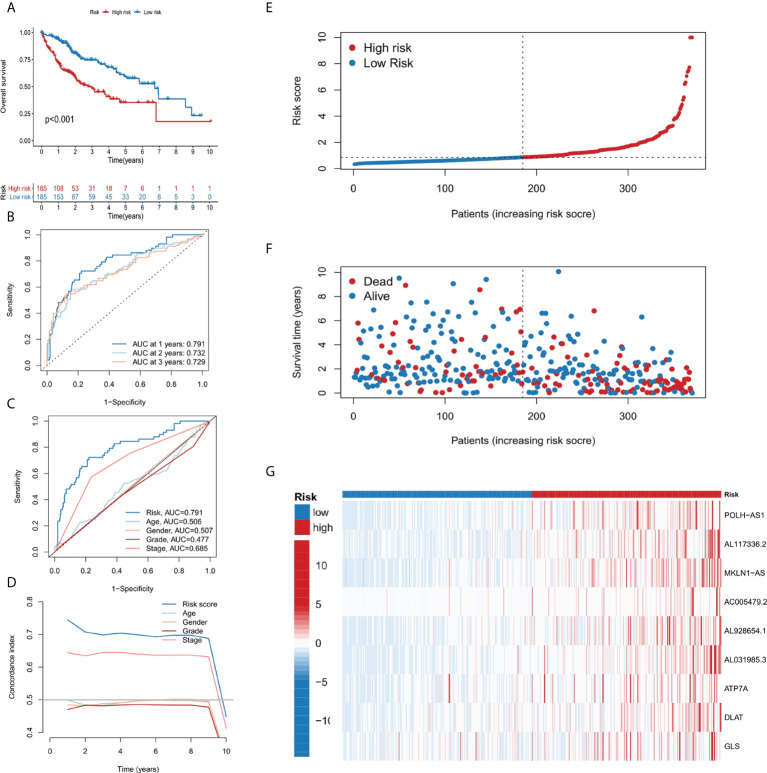
Establishment and evaluation of the 9-gene signature. **(A)** Kaplan-Meier survival analysis of liver cancer patients in the high-risk and low-risk groups. **(B)** Time dependent ROC curves of overall survival at 1, 2- and 3- years. **(C)** ROC curves of risk score for clinical features in liver cancer patients. **(D)** The C-index curve analyzes the consistency index of risk score. **(E–G)** The distribution of risk score, survival status, and 9-gene expression profiles for each liver cancer patient.

Patients in the TCGA cohort were divided into low-risk and high-risk subgroups according to the median risk score. The overall survival rate (OS) of low-risk group was significantly lower than that of high-risk group (**Figure 3A**). In order to study the prediction accuracy of this 9-gene signature, we conducted a time-dependent ROC analysis (**Figures 3B, C**). Besides, compared with other variables, the risk score (AUC = 0.791) was a better predictor than other clinical traits, such as age (AUC = 0.506), gender (AUC = 0.507), grade (AUC = 0.477) and stage (AUC = 0.685). The area under the ROC curve (AUC) of OS was 0.791 at 1 year, 0.732 at 2 years and 0.729 at 3 years, indicating the 9-gene signature had a good prognostic prediction efficacy. Next, we constructed a C-Index curve of risk score and clinical traits (age, gender, and stage), and the results showed the consistency of risk score was higher than that of the clinical traits (**Figure 3D**). In addition, the distribution of patient risk score, survival status and the 9 genes expression profiles were shown in **Figures 3E–G**.

### Construction and validation of the nomogram

**Figure 4:**
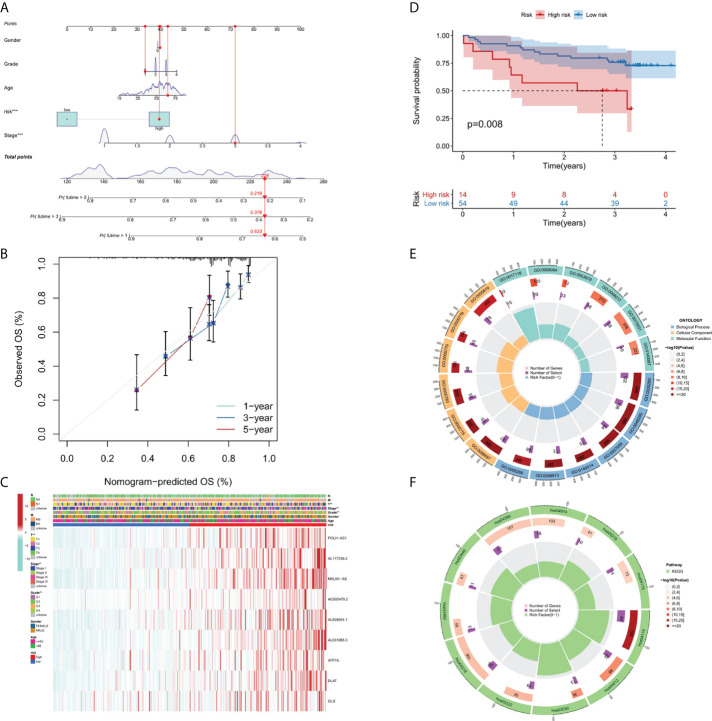
Nomogram, calibration curves and functional enrichment analysis. **(A)** Nomograms predicting 1-year, 3-year and 5-year OS for patients with liver cancer. **(B)** Nomogram model calibration curve. **(C)** Heatmap displaying expression profile of the 9 genes and correlation between clinical features and risk score. **(D)** Results of Kaplan-Meier analysis for the different RS score groups in GEO dataset. **(E)** Results of GO enrichment analyses. **(F)** Results of KEGG enrichment analyses.

A nomograph model including risk score, age, gender, grade, and stage was constructed to predict the 1-, 3- and 5-years OS of liver cancer patients by calculating the nomograph score based on the point scale (**Figure 4A**). The calibration curve of nomogram showed that there was a good consistency between the predicted results and the observed results (**Figure 4B**).

### The correlation between risk score and different clinicopathological factors

In order to clarify the clinical significance of risk score, the correlation between risk score and major clinicopathological variables such as gender, age, grade, and pathological stage were analyzed. As showcased in **Figure 4C**, the clinical grade and T stage of liver cancer patients in the high-risk group was later than that in the low-risk group, suggesting a worse prognosis than the low-risk group.

### Validation of cuproptosis-related risk model in GEO dataset

Since there are no corresponding lncRNAs in other data sets, it is difficult to verify the performance of the risk model. Therefore, we calculated the RS scores in TCGA and GSE144269. There was a strongly correlation between risk score and RS score in TCGA dataset (p-value = 1.016 * 10^-5^), indicating that the RS score can be used as an alternative scoring model for risk score. The result of Kaplan–Meier (KM) survival analysis showed the 9-gene signature exhibited good prognostic performance (**Figure 4D**).

### Functional enrichment analysis

We performed GO (**Figure 4E**) and KEGG (**Figure 4F**) pathway analysis on genes of differentially expressed lncRNAs and mRNAs in two risk groups. In BP (biological processes) category, cell division related activity including nuclear division (GO:0000280), organelle fission (GO:0048285) and chromosome segregation (GO:0007059) were significantly enriched. In CC (cellular components) category, the differentially expressed genes were involved in chromosomal region (GO:0098687), centromeric region (GO:0000775), condensed chromosome (GO:0000793), kinetochore (GO:0000776) and spindle (GO:0005819). In MF (molecular functions) category, DEGs were enriched in enriched in DNA-related activities, such as single-stranded DNA helicase activity (GO:0017116), ATP-dependent activity (GO:0008094) and DNA helicase activity (GO:0003678). KEGG pathway analysis showed that differentially expressed genes were mainly enriched in Cell cycle (hsa04110), DNA replication (hsa03030), and p53 signaling pathway (hsa04115), etc. In summary, the results of the enrichment analysis showed the 9-gene signature was closely associated with cell proliferation.

### Immune-related functional analysis

**Figure 5:**
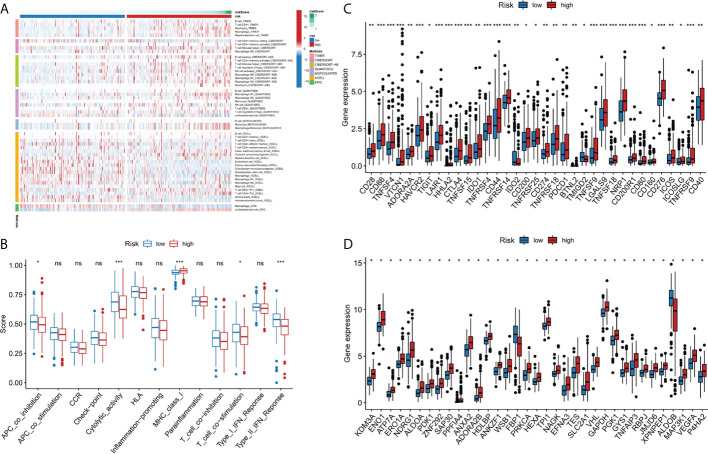
Immune-related functional analysis. **(A)** The landscape of immune infiltration in two risk groups for liver cancer patients. **(B)** Boxplot visualizing differentially immune functions. “ns” stands for not significant. **(C)** Comparisons of immune checkpoints between the two risk groups in liver cancer patients. *p < 0.05; **p < 0.01; ***p < 0.001. **(D)** Comparison of the expression of hypoxia-associated genes between the two risk groups in liver cancer patients. *p < 0.001. ns, not significant.

Immune cell infiltration is a crucial component of tumor microenvironment, which is strongly related to tumor behavior and patient prognosis (22, 23). Several algorithms (TIMER, XCELL, QUANTISEQ, MCPCOUNTER, CIBERSORT, CIBERSORT-ABS and EPIC) were used to study the correlation between the infiltration of various immune cells and risk score and the results were shown in **Figure 5A**. In the ssGSEA, the immune status of low-risk group was relatively higher than that of high-risk group (**Figure 5B**). In addition, we found that the high risk group showed a higher expression level of immune checkpoint genes, thus indicating a better response to immunotherapy (**Figure 5C**). In conclusion, these results suggested that the 9-gene signature was related to immune cell infiltration to some extent.

### Relationship between hypoxia-related gene and risk score

Since there may be a close relationship between hypoxia and cuproptosis, we studied the expression of hypoxia related genes in the two risk groups. The majority of the hypoxia-related genes, such as LXN, HAS1, AKAP12, and ETS1, were significant upregulated in the high risk group than in the low risk group (p<10^-10^) (**Figure 5D**). Based on the above results, we considered there were relations between cuproptosis and hypoxia in liver cancer, and specific clinical trials are needed, to further test this hypothesis.

### Analysis of tumor mutation burden and drug sensitivity

**Figure 6:**
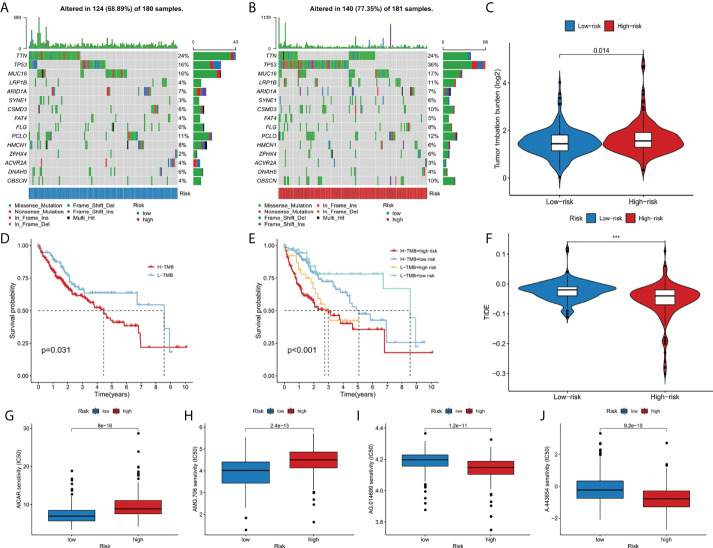
The relationship between TMB and the 9-gene signature. **(A, B)** The oncoplots of the mutation genes in liver cancer patients for the high-risk and low-risk groups. **(C)** Higher TMB levels correlated with high-risk group. **(D)** Higher TMB level demonstrated poorer OS. **(E)** Kaplan-Meier curves for patients by both risk score and TMB. **(F)** Higher TIDE levels correlated with low-risk group. **(G-J)** Drug sensitivity analysis. ***p < 0.001.

The somatic mutation data of liver cancer patients were downloaded from the TCGA database and visualized using the “maftools” R software package. The results showed the mutation frequency in high-risk group was higher than that in low-risk group (77.35% vs. 68.89%), and in high risk group, mutation rate of TP53 was dramatically higher than that in low risk group (36% vs 16%) (**Figures 6A, B**). In addition, we studied the difference of tumor mutation burden between high-risk group and low-risk group, and the result showed the high risk score group had significant higher tumor mutation burden than the low risk group (p<0.05) (**Figure 6C**). As shown in the **Figure 6D**, the high TMB group had a better prognosis than low TMB group. Then, we further combined risk score and TMB to evaluate the prognosis of liver cancer patients, and we found the overall survival rate of low TMB and low-risk group was the best, and that of high TMB and high-risk group was the worst (**Figure 6E**). In addition, the sensitivity difference of immunotherapy between high-risk group and low-risk group was further studied based on TIDE algorithm. We found that the TIDE level was higher in the low-risk group than in the high-risk group, indicating that patients in the high-risk group had a lower possibility of immune escape, and better immunotherapy (**Figure 6F**). Finally, we used the R software package pRRophetic to analyze the half maximum inhibitory concentration (IC50) of some chemotherapeutic drugs commonly in two risk groups. AICAR (**Figure 6G**) and AMG.706 (**Figure 6H**) were more effective in the low-risk group, while AG.014699 (**Figure 6I**) and A.443654 (**Figure 6J**) were more efficacious in the high-risk group (p<10^-11^).

At last, we attempted to compare our 9-gene signature with other previously published cuproptosis-related prognostic models in liver cancer, but there is rare paper in this field. Our model may initiate research in this field.

## Discussion

Copper is an essential mineral for human health (24). The fact that dysregulation of copper level will cause damage to the normal cell life has been known for a long time. The discovery of cuproptosis makes an interesting and important explanation for this phenomenon (25). Although the field of cuproptosis is just in its infancy, it is reasonable to speculate that this cell death mode has tight correlation with human diseases, including liver cancer. There are two basic elements for the initiation of cuproptosis: copper and lipoylated protein. About the first element, liver is the central organ for the metabolism of copper (26, 27). For the lipoylated proteins, most of them function in the TCA cycle in the mitochondrion. To avoid cuproptosis, these two elements must be maintained in check. In liver cancer cell, dysregulation of the normal gene regulatory network is a major hallmark, which applies to the essential genes associated with copper metabolism and TCA cycle (28, 29). It will not be surprising that liver cancer cell may be one of the major sites where cuproptosis happens. We believe that in the near future, the regulation and the pathologic relevance of cuproptosis will be revealed in liver cancer. Before that, if we can utilize the key genes in cuproptosis to make a prognostic model for liver cancer patient, this will accelerate the translation of this field to clinical practice, which also applies to other tumor types (30).

Given the severe threat of liver cancer to human society, much more efforts are needed to figure out the etiology and pathogenesis of liver cancer and develop effective therapeutics to treat it. In the meantime, advances in the diagnosis and prognosis techniques for liver cancer are of urgent demand. In this study, we leveraged the lncRNA and mRNA associated with cuproptosis to make a novel prognostic signature for liver cancer. In this model, we incorporated 6 lncRNAs (POLH-AS1, AL117336.2, MKLN1-AS, AC005479.2, AL928654.1, and AL031985.3) and 3 mRNAs (ATP7A, DLAT, and GLS) after a stringent selection process (**Figures 1**, **2**). Our model exhibits an effective application in the prognosis of liver cancer patient (**Figures 3**, **4**). Our model is capable of reflecting several major hallmarks (including the immune cell infiltration and TMB) of liver cancer (**Figures 5**, **6**). This model also satisfactorily passed the validation procedure. The inclusion of the 3 mRNAs is an advantage of our model compared with those only analyze lncRNA. These proteins encoded by the 3 mRNAs are closely related to the induction of cuproptosis. ATP7A is a copper exporter, which is essential for the homeostasis of intracellular copper level (31). Mutation of ATP7A is demonstrated to be associated with Menkes disease, occipital horn syndrome, and X-linked distal spinal muscular atrophy (32). It will be interesting to investigate whether there is liver cancer-related mutation in ATP7A gene and if so, whether this mutation will affect cuproptosis and liver cancer development. DLAT is an important subunit of the PDH complex and also one of the major substrates for cuproptosis (33). A recent study found that the DLAT level in hepatocellular carcinoma was influenced by blueberry malvidin-3-galactoside and 5-fluorouracil (34). This may establish links between cuproptosis and intestinal microbiota and chemotherapy of liver cancer. GLS, as a mitochondrial glutaminase, which hydrolyzes glutamine to glutamate, has a close relationship with liver cancer progression (35, 36). Huang et al. revealed that GLS along with PDH complex was important in liver cancer metabolism and autophagy, which might be the underlying mechanism for the chemo-resistance of liver cancer cell (37). Whether cuproptosis participates in the regulation of sensitivity to chemotherapy of liver cancer cell needs to be clarified in the future. For these lncRNAs, several of them have been linked with other RCDs like ferroptosis (POLH-AS1, MKLN1-AS, AL928654.1, and AL031985.3) and pyroptosis (MKLN1-AS, AC005479.2, and AL031985.3) in liver cancer (38–41). Their involvement with cuproptosis suggests there may be molecular relevance between those distinct RCDs. In fact, these RCDs do share similar mechanism of initiation (for example, ROS triggers apoptosis and ferroptosis), regulator (like Caspases in apoptosis, pyroptosis, and necroptosis; metal ion in ferroptosis and cuproptosis), and function (for example, apoptosis, ferroptosis, and pyroptosis all modulate immune activity) (10, 11). We wish future researches will shed more light on the link between these different RCDs. Moreover, POLH-AS1, AC005479.2 and AL928654.1 have been found to involve immune response in papillary thyroid cancer and hepatocellular carcinoma, respectively (42–44). Our model not only incorporates these two lncRNAs, but also expand this list of immune-associated lncRNAs in liver cancer to other 4 lncRNAs. AL117336.2 is a novel lncRNA with little study to date. It is worthy to explore its potential role in liver cancer and cuproptosis in the future. As hypoxia can dramatically influence TCA cycle and mitochondrion function, our results may reveal a link between hypoxia and cuproptosis in liver cancer (**Figure 5D**) (21). Another interesting result about our model is its correlation with TMB in liver cancer, which is related to the sensitivity of liver cancer cell to several chemotherapeutic drugs (**Figure 6**). A notable point is the mutual exclusiveness or co-occurence of p53 mutation and mutations of other genes. p53 here is of vital pathological relevance, not only because it is among the most important tumor suppressor genes, but also that it is a master regulator of several RCDs, including apoptosis, ferroptosis, and pyroptosis (45–47). p53 also has vital role in regulating TCA cycle (45). It should be one major direction to study whether p53 can regulate cuproptosis or not in liver cancer.

To sum up, we established an effective prognostic signature in liver cancer based on cuproptosis-related lncRNAs and mRNAs. We believe that this work will not only benefit the liver cancer patient in clinical use, but also make useful suggestions for the research field of cuproptosis. We wish in the near future, there will be great advances in researches about cuproptosis in liver cancer, based on which we can validate and improve our model to make it more accurate and efficient.

## Data availability statement

The original contributions presented in the study are included in the article/**Supplementary Material**. Further inquiries can be directed to the corresponding author. The public datasets analyzed in this study can be found in the TCGA (https://portal.gdc.cancer.gov/) and GEO (https://www.ncbi.nlm.nih.gov/geo/) repository.

## Author contributions

LM and YQL conceived the project. YL, YQL, and HF analyzed the data. YL, YQL, SY, and HF wrote the manuscript. LM reviewed and revised the manuscript. The authors read and approved the final manuscript. The requirements for authorship have been met. Each author believes that the manuscript represents honest work.

## Funding

This work was supported by grants from the Natural Science Foundation of Jilin Province, China [20210101248JC].

## Conflict of interest

The authors declare that the research was conducted in the absence of any commercial or financial relationships that could be construed as a potential conflict of interest.

## Publisher’s note

All claims expressed in this article are solely those of the authors and do not necessarily represent those of their affiliated organizations, or those of the publisher, the editors and the reviewers. Any product that may be evaluated in this article, or claim that may be made by its manufacturer, is not guaranteed or endorsed by the publisher.
